# Mr. Wardrop's Morbid Anatomy of the Human Eye

**Published:** 1835-01-01

**Authors:** 


THE MORBID ANATOMY OF THE HUMAN EYE. By *James Wardrop*, Surgeon to the late King. Illustrated by coloured-plates. Second edition. Two vols. 8vo. pp. 175—263. Plates XVIII. Churchill, London, 1834.

The following advertisement to this edition is seldom presented to the public by authors.

ADVERTISEMENT.

"Nothing would have prevented the Author from supplying the demand which has for some years past been made for this work, but difficulties in getting the plates as accurately coloured as in the former edition, and he embraces this opportunity of remarking, that, although twenty-six years have elapsed since the Morbid Anatomy of the Eye was first published, the care with which he selected the materials is satisfactorily proved by the circumstance, that subsequent researches in this interesting department of Pathology have not contributed any additional facts to render any alteration in the work desirable."

This statement, if correct, and no doubt it is so, constitutes no common praise. Yet while it displays the merits of the work it precludes a more circumstantial notice of its contents. We will only add, that the precision of detail displayed in the writing, is equalled by the clearness of engraving and of colouring exhibited in the plates. Probably the majority of well-informed surgeons are already in possession of the first edition. Those of our readers who are not, will do well to make themselves possessors of the present.

